# Anaplastic Large Cell Lymphoma of the Brain Mimicking an Extra-Axial Frontal Tumor: A Case Report

**DOI:** 10.7759/cureus.117242

**Published:** 2026-09-30

**Authors:** Seema V Vishwakarma, Rahul Rajbhar, Shilpi Sahu

**Affiliations:** 1 Department of Pathology, Mahatma Gandhi Mission's Medical College, Navi Mumbai, IND

**Keywords:** anaplastic large-cell lymphoma, brain, extra-axial lesions, herniation, lymphoma, meningioma, t-cell

## Abstract

Primary central nervous system anaplastic large cell lymphoma (PCNS-ALCL) is an extremely rare subtype of T-cell lymphoma with variable clinicoradiological presentation and significant diagnostic challenges. Extra-axial dural-based lesions frequently mimic meningioma radiologically, often leading to delayed diagnosis. We report a case of a 41-year-old female presenting with severe headache followed by sudden-onset altered sensorium. Neuroimaging revealed a lobulated hyperdense extra-axial frontal lesion associated with extensive vasogenic edema, ventricular compression, and marked midline shift suggestive of aggressive intracranial pathology. Emergency surgical excision was performed because of significant mass effect and impending herniation. Histopathological examination demonstrated diffuse infiltration of the brain parenchyma by pleomorphic plasmacytoid tumor cells with marked atypia, brisk mitotic activity, and focal necrosis. Immunohistochemistry showed diffuse positivity for cluster of differentiation (CD)30, leukocyte common antigen (LCA), and epithelial membrane antigen (EMA) with a high Ki-67 proliferative index, while tumor cells were negative for glial fibrillary acidic protein (GFAP), pan-cytokeratin (PanCK), CD20, CD3, CD10, CD138, spalt-like transcription factor 4 (SALL4), and anaplastic lymphoma kinase (ALK)-1. Based on histomorphology and immunophenotypic profile, a final diagnosis of PCNS ALK-negative ALCL was established. This case highlights the importance of considering lymphoma in the differential diagnosis of rapidly progressive extra-axial lesions associated with disproportionate edema and acute neurological deterioration.

## Introduction

Primary central nervous system lymphoma (PCNSL) is a rare extranodal non-Hodgkin lymphoma confined to the brain, spinal cord, leptomeninges, or eyes without systemic involvement at diagnosis [1,2]. It constitutes approximately 2-3% of all primary intracranial neoplasms and nearly 4-6% of extranodal lymphomas [3]. Diffuse large B-cell lymphoma accounts for the majority of PCNSLs, whereas T-cell lymphomas represent less than 5% of cases [4]. Among these rare T-cell neoplasms, primary central nervous system (PCNS) anaplastic large cell lymphoma (ALCL) is exceptionally uncommon. It remains one of the least frequently encountered entities in neuropathology practice [5,6].

ALCL is a mature T-cell neoplasm characterized by diffuse proliferation of large pleomorphic lymphoid cells with strong and uniform CD30 expression [7]. According to the WHO Classification of Haematolymphoid Tumours, ALCL is classified into anaplastic lymphoma kinase (ALK)-positive ALCL, ALK-negative ALCL, primary cutaneous ALCL, and breast implant-associated ALCL. ALK-positive tumors generally occur in younger individuals and are associated with a relatively favorable prognosis. In contrast, ALK-negative tumors occur predominantly in adults and demonstrate aggressive biological behavior with poorer clinical outcomes [8].

PCNS ALCL presents significant diagnostic difficulty because of nonspecific clinical manifestations and highly variable radiological appearance. Patients commonly present with headache, vomiting, seizures, altered sensorium, focal neurological deficits, or signs of raised intracranial pressure [9,10]. Radiologically, these tumors may appear intra-axial or extra-axial and frequently mimic meningioma, metastatic tumors, glioblastoma, or other aggressive intracranial neoplasms [5].

We report a rare case of primary CNS ALK-negative ALCL presenting as a rapidly progressive extra-axial frontal tumor radiologically mimicking meningioma. The present case highlights the importance of clinicoradiological suspicion and comprehensive histopathological evaluation in diagnosing unusual aggressive CNS lymphomas.

## Case presentation

A 41-year-old female patient presented with complaints of severe headache for 20 days, followed by sudden-onset altered sensorium for two days. There was no history of seizures, trauma, visual disturbances, focal neurological deficit, or known systemic malignancy. The patient had progressive deterioration in sensorium associated with increasing drowsiness and reduced responsiveness. Clinical examination revealed altered mental status with signs suggestive of raised intracranial pressure. In view of acute neurological deterioration, urgent neuroimaging was advised.

Non-contrast multidetector computed tomography (MDCT) of the brain revealed diffuse effacement of sulci in bilateral cerebral hemispheres, suggestive of cerebral edema (Figure 1).

**Figure 1:**
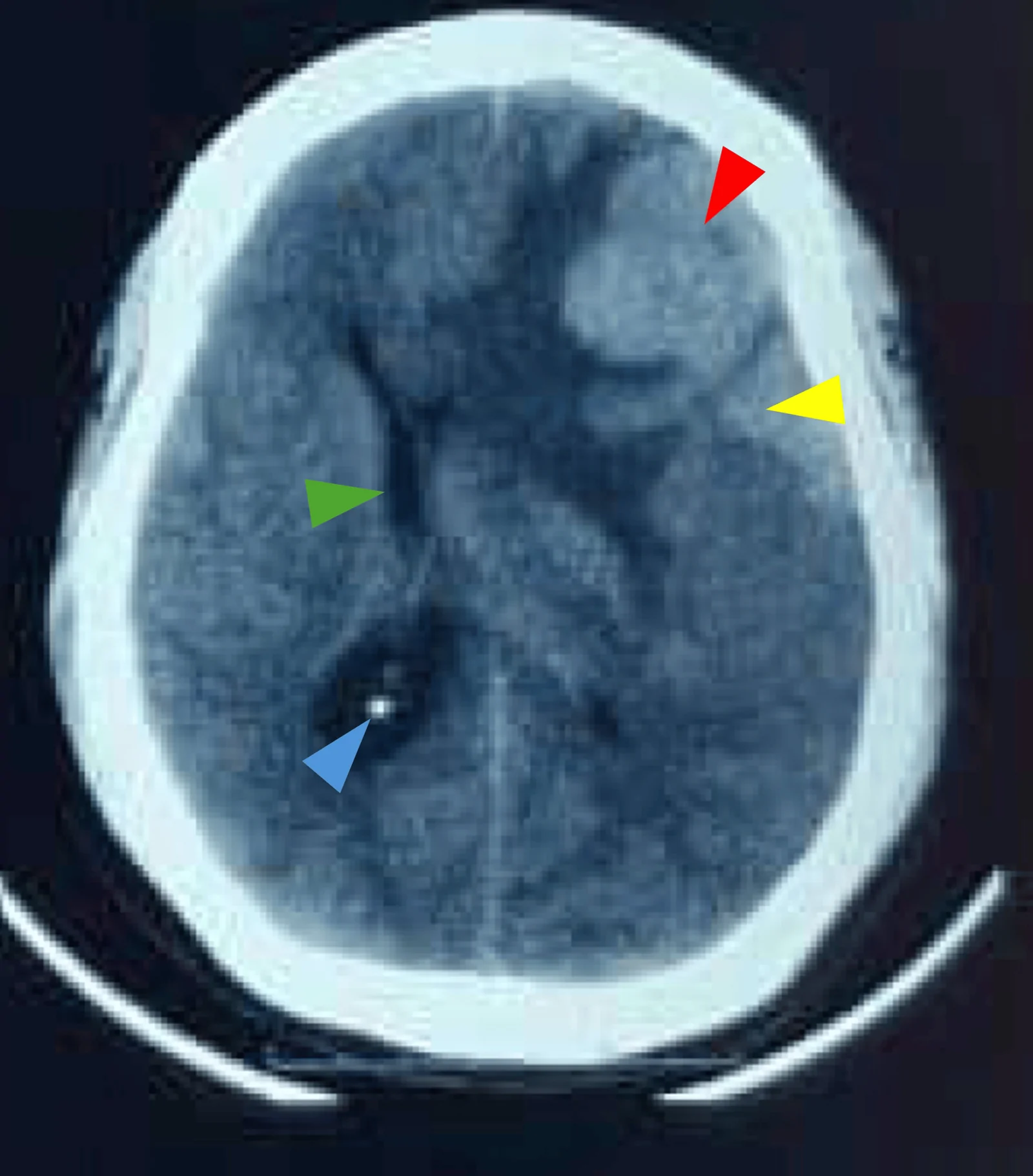
Axial non-contrast computed tomography scan of the brain showing a lobulated hyperdense extra-axial lesion in the left frontal region with a broad base toward the dura and extensive surrounding vasogenic edema The red arrow indicates a lobulated hyperdense extra-axial lesion (left frontal region). The yellow arrow indicates surrounding vasogenic edema. The blue arrow indicates compression of the left lateral ventricle. The green arrow indicates the midline shift towards the right.

A lobulated hyperdense lesion with an average attenuation of approximately 42 HU measuring 3.7 × 5.6 × 3.4 cm was identified in the left frontal region. The lesion appeared extra-axial with broad-based dural attachment. Extensive vasogenic edema was noted involving the left frontotemporal lobes. Significant mass effect was present in the form of compression of the left lateral ventricle, diffuse sulcal effacement, subfalcine herniation, and a midline shift of approximately 1.2 cm toward the right side. Posterior fossa structures and the brainstem appeared unremarkable. Based on imaging findings, an extra-axial neoplastic lesion was suspected, with meningioma and metastatic tumor considered as primary radiological differentials. However, the extensive edema, marked mass effect, and rapid neurological deterioration were atypical for benign meningioma and favored aggressive pathology.

In view of the significant mass effect and impending herniation, the patient underwent a left fronto-temporo-parietal craniotomy with gross total excision of the tumor. Intraoperatively, the tumor appeared grayish-white, soft to firm in consistency, and moderately vascular. The lesion involved the left frontal lobe with extension toward the temporal region and sylvian fissure. Gross total excision was achieved, and adequate hemostasis was secured (Figure 2).

**Figure 2:**
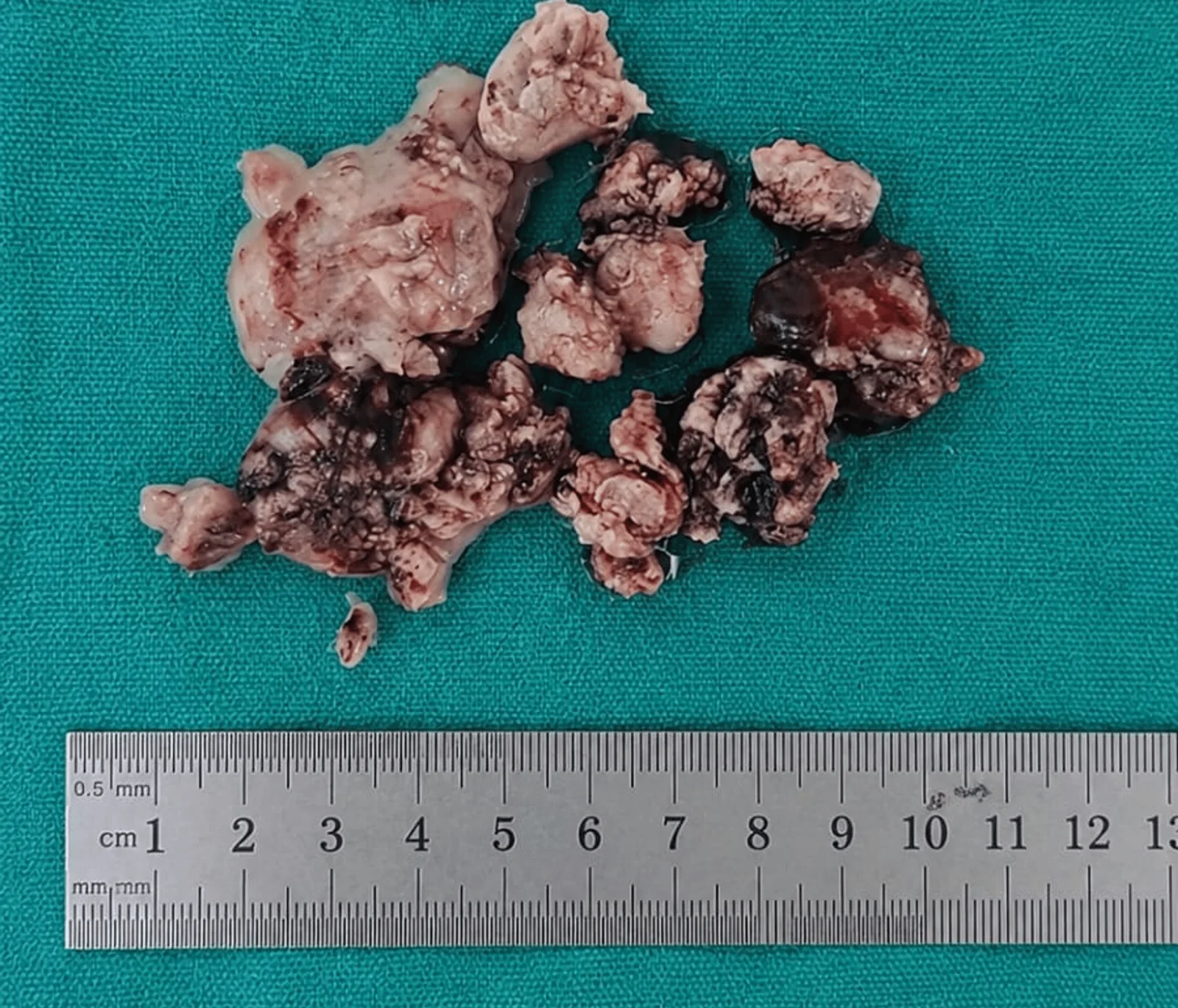
Gross specimen showing multiple grey-white to grey-brown friable tissue fragments aggregating to 8 x 4 x 1 cm

The excised specimen was sent for histopathology and immunohistochemistry. Postoperatively, the patient was monitored in the neurosurgical intensive care unit with serial neurological assessment and repeat neuroimaging.

Microscopic examination of multiple fragmented tissue bits revealed diffuse infiltration of the brain parenchyma by highly pleomorphic tumor cells arranged predominantly in sheets with focal nests and lobular architecture. Individual tumor cells were round to oval to plasmacytoid in morphology and showed increased nuclear-to-cytoplasmic ratio, vesicular chromatin, prominent macro-nucleoli, and scant cytoplasm. Many cells exhibited deeply basophilic cytoplasm with angulated nuclei and eosinophilic cytoplasm. Marked nuclear pleomorphism, numerous atypical mitotic figures, and occasional bizarre tumor giant cells were identified. Focal areas of tumor necrosis were noted along with dilated and congested blood vessels at the periphery (Figure 3).

**Figure 3:**
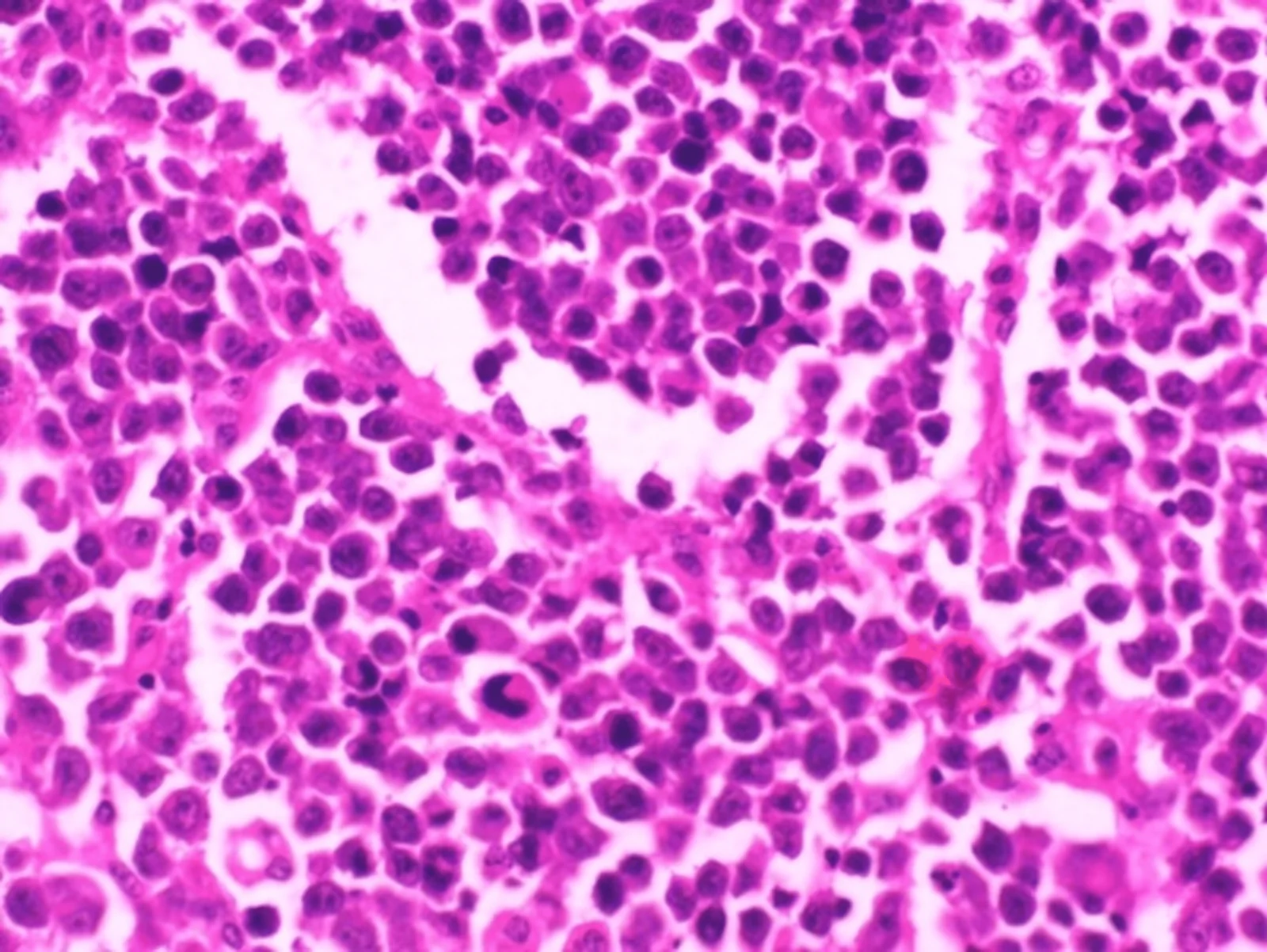
Hematoxylin and eosin stained section, showing pleomorphic tumor cells infiltration (400X)

The histomorphological features were suggestive of a high-grade malignant neoplasm, with high-grade hematolymphoid neoplasm and high-grade epithelial metastasis considered as the principal differential diagnoses. Immunohistochemistry was performed for further characterization of the tumor.

Tumor cells demonstrated diffuse strong positivity for leukocyte common antigen (LCA), CD30, and epithelial membrane antigen (EMA). The Ki-67 labeling index was approximately 50%, indicating highly aggressive proliferative activity. Tumor cells were negative for glial fibrillary acidic protein (GFAP), pan-cytokeratin (PanCK), CD20, CD3, CD10, CD138, spalt-like transcription factor 4 (SALL4), and ALK-1. The absence of GFAP excluded high-grade glial neoplasm, while negativity for PanCK and SALL4 helped rule out metastatic carcinoma and germ cell tumor respectively. Based on histomorphology and immunophenotypic profile, the final diagnosis was established as PCNS ALK-negative ALCL (Figure 4).

**Figure 4:**
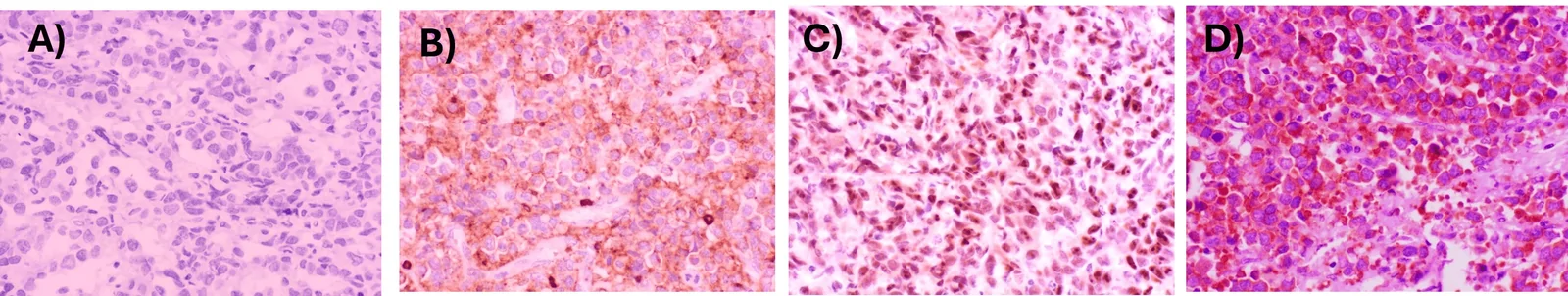
immunostained sections A) ALK-1 immunostain negative in tumor cells (400X); B) EMA immunostain showing membranous positivity in tumor cells (400X); C) Ki-67 immunostain showing a high proliferative index (50%) in tumor cells (400X); D) CD30 immunostain showing strong positivity in tumor cells (400X). ALK: anaplastic lymphoma kinase; EMA: epithelial membrane antigen; CD: cluster of differentiation.

Additional molecular investigations, including the next-generation sequencing (NGS) lymphoma panel, dual specificity phosphatase 22 and interferon regulatory factor 4 (DUSP22::IRF4) rearrangement analysis, tumor protein p53 (TP53) mutation analysis, and T-cell receptor gene rearrangement studies, were advised for further subclassification and prognostic evaluation. However, these investigations could not be performed due to the patient’s financial constraints.

The patient was subsequently transferred to another hospital, where conservative management was pursued. She was thereafter lost to follow-up at our institution; however, subsequent communication with her relatives revealed that she had died.

## Discussion

ALCL is a mature T-cell neoplasm characterized by large pleomorphic lymphoid cells and strong CD30 expression. Clinically, patients with PCNS ALCL may present with nonspecific symptoms such as headache, vomiting, seizures, focal neurological deficits, altered sensorium, or features of raised intracranial pressure. The present patient had a severe headache for 20 days, followed by a sudden onset of altered sensorium for two days. This presentation suggests rapid disease progression with raised intracranial pressure. Similar clinical features have been reported by Hirano et al. and Brady et al., where patients with PCNS ALCL presented with headache, neurological deficits, and rapid deterioration [10,11]. Compared with those reports, the present case showed a more acute presentation due to significant cerebral edema and midline shift.

Radiologically, the present case showed a lobulated hyperdense extra-axial lesion in the left frontal region with broad-based dural attachment, extensive vasogenic edema, compression of the left lateral ventricle, subfalcine herniation, and midline shift. These findings initially suggested meningioma or metastasis. However, the degree of edema and acute neurological deterioration were disproportionate for a benign meningioma and favored aggressive pathology. Inami et al. reported a similar meningioma-like dural lymphoma, where the lesion appeared extra-axial with broad tentorial attachment and extensive edema, initially mimicking meningioma. Their case also highlighted that rapid growth and atypical imaging findings should raise suspicion for lymphoma rather than benign meningioma [12]. This comparison is important because both lesions were dural-based and radiologically mimicked meningioma. However, Inami et al. reported a diffuse large B-cell lymphoma, whereas the present case is ALK-negative ALCL, a much rarer T-cell lymphoma. Both cases demonstrate that lymphoma can present as an extra-axial lesion and should be included in the differential diagnosis when imaging shows hyperdensity, extensive edema, rapid clinical progression, and mass effects.

Histopathologically, the present tumor demonstrated marked pleomorphism with a prominent plasmacytoid morphology, brisk atypical mitotic activity, bizarre tumor cells, and focal necrosis. These findings showed considerable overlap with other high-grade intracranial malignancies. Stein et al. described diffuse sheets of large pleomorphic lymphoid cells with prominent nucleoli and brisk mitotic activity as characteristic morphological features of ALCL [13]. Similarly, Yim et al. reported marked cellular atypia and aggressive histomorphological features in PCNS T-cell lymphomas [14]. Compared with previously reported cases, the prominent plasmacytoid morphology, extensive pleomorphism, and necrosis in the present case further complicated the interpretation of routine histology. Poorly differentiated metastatic carcinoma may demonstrate sheets of pleomorphic cells with necrosis; glioblastoma may show marked atypia, mitotic activity, and necrosis; plasmablastic lymphoma may exhibit prominent plasmacytoid morphology; and melanoma may also present as a highly pleomorphic malignant neoplasm. Therefore, morphology alone was insufficient for definitive classification, necessitating immunohistochemical evaluation for accurate lineage determination. Similar diagnostic difficulty was described in CNS lymphoma cases, where lymphoma may mimic other malignant CNS tumors on morphology and imaging [14].

Immunohistochemistry played the most important role in establishing the diagnosis. The tumor cells were positive for LCA, confirming hematolymphoid origin. Strong CD30 positivity supported ALCL, while EMA positivity further favored ALCL. The Ki-67 labeling index was approximately 50%, indicating high proliferative activity. Tumor cells were negative for GFAP, excluding high-grade glial tumor; negative for PanCK, excluding metastatic epithelial malignancy; negative for CD20, excluding B-cell lymphoma; negative for CD138, arguing against plasma cell neoplasm or plasmablastic lymphoma; and negative for SALL4, excluding germ cell tumor. ALK-1 negativity classified the tumor as ALK-negative ALCL [11,13].

The immunohistochemistry profile of the present case also differs from the lymphoma case reported by Inami et al. In their report, tumor cells were CD20 positive and negative for CD3, AE1/3, and GFAP, supporting diffuse large B-cell lymphoma. In contrast, the present case was CD30, EMA, and LCA positive but CD20 negative, confirming a T-cell lymphoma phenotype consistent with ALCL [12]. Thus, both cases highlight the same principle: radiology may suggest meningioma, and morphology may suggest high-grade malignancy, but immunohistochemistry is essential for exact lineage determination.

ALK status has major prognostic significance in ALCL. ALK-positive tumors generally show better response to therapy and improved survival, whereas ALK-negative tumors are associated with an aggressive clinical course. Morrow et al. identified age and ALK status as important prognostic factors in primary CNS ALCL. The present case was ALK-negative and showed aggressive features, including sudden neurological decline, marked edema, midline shift, necrosis, and a high Ki-67 index. These findings are in concordance with previous reports suggesting that ALK-negative CNS ALCL behaves more aggressively than ALK-positive disease [10,15].

The Ki-67 index in the present case was approximately 50%, supporting aggressive biological behavior. High Ki-67 is commonly associated with rapid tumor growth, high mitotic activity, and adverse prognosis. Brady et al. described high proliferative activity in PCNS ALK-negative ALCL, while Hirano et al. also observed that ALK-negative cases often show aggressive behavior. In the present case, the high Ki-67 correlated with the radiological findings of extensive edema and clinical findings of acute altered sensorium [10,11].

Molecular studies are increasingly important in ALK-negative ALCL. Genetic alterations such as DUSP22 rearrangement, TP63 rearrangement, TP53 deletion, and janus kinase/signal transducer and activator of transcription (JAK/STAT) pathway activation may influence prognosis and therapeutic response [16]. In the present case, NGS lymphoma panel, DUSP22::IRF4 rearrangement study, TP53 mutation analysis, and T-cell receptor gene rearrangement were advised. Yim et al. emphasized the value of molecular profiling in PCNS T-cell lymphomas [14]. Molecular testing may therefore help in further subclassification, prognostication, and future targeted therapy selection.

Management of PCNS ALCL is difficult because of its rarity and lack of standardized treatment guidelines. In the present case, surgical excision was required because of severe mass effect, midline shift, and acute neurological deterioration. However, surgery alone is not adequate for lymphoma because of its infiltrative and systemic biological behavior. High-dose methotrexate-based chemotherapy with or without radiotherapy is generally considered the main therapeutic approach for CNS lymphomas. Hirano et al. reported better outcomes in patients treated with multimodal therapy. Inami et al.’s dural lymphoma case also showed marked tumor regression after chemotherapy, supporting the chemosensitive nature of CNS lymphomas [10,12,17].

Overall, the present case emphasizes that lymphoma should be considered in the differential diagnosis of any rapidly progressive extra-axial mass lesion showing disproportionate vasogenic edema, hyperdensity on CT, acute neurological deterioration, and marked mass effect. Although meningioma is the most common extra-axial tumor, not all dural-based lesions are meningiomas. A broad immunochemistry panel is necessary to differentiate ALCL from metastatic carcinoma, high-grade glioma, germ cell tumor, B-cell lymphoma, plasmablastic lymphoma, and plasma cell neoplasm [11,12].

Thus, this case contributes to the limited literature on PCNS ALK-negative ALCL and expands the spectrum of meningioma-mimicking CNS lymphomas. Early recognition, prompt biopsy or excision, comprehensive immunohistochemistry, and appropriate multimodal treatment are essential for improving patient outcome. Awareness of this rare entity is important for neurosurgeons, radiologists, pathologists, and oncologists because timely diagnosis directly influences treatment planning and prognosis [10,15].

## Conclusions

This case underscores the importance of a multidisciplinary approach involving radiology, neurosurgery, and pathology in the evaluation of intracranial tumors. Extra-axial brain lesions with atypical imaging features, disproportionate vasogenic edema, and rapid neurological deterioration should raise suspicion for aggressive malignancies other than meningioma. Histopathological examination and a broad immunohistochemical panel are essential for establishing tumor lineage and excluding major diagnostic mimics. Strong CD30, LCA, and EMA positivity with ALK-1 negativity supported the diagnosis of ALK-negative ALCL in this case. Because comprehensive systemic staging and molecular studies were not available, the designation of PCNS disease should be interpreted in the context of the available clinical information. Awareness of this rare meningioma-mimicking presentation may facilitate earlier recognition and appropriate multidisciplinary management.
